# Potential and Therapeutic Roles of Diosmin in Human Diseases

**DOI:** 10.3390/biomedicines10051076

**Published:** 2022-05-06

**Authors:** Etimad Huwait, Mohammad Mobashir

**Affiliations:** 1 Department of Biochemistry, Faculty of Sciences, King Abdulaziz University, Jeddah 22254, Saudi Arabia; 2 Cell Culture Lab, Experimental Biochemistry Unit, King Fahad Medical Research Centre, King Abdulaziz University, Jeddah 22252, Saudi Arabia; 3 SciLifeLab, Department of Oncology and Pathology, Karolinska Institutet, P.O. Box 1031, 17121 Stockholm, Sweden; 4 Genome Biology Lab, Department of Biosciences, Faculty of Natural Science, Jamia Millia Islamia, New Delhi 110025, India

**Keywords:** Diosmin, target pathways, anti-inflammatory, anti-oxidant, anti-cardiovascular, anti-diabetic, anti-cancer

## Abstract

Because of their medicinal characteristics, effectiveness, and importance, plant-derived flavonoids have been a possible subject of research for many years, particularly in the last decade. Plants contain a huge number of flavonoids, and Diosmin, a flavone glycoside, is one of them. Numerous *in-vitro* and *in-vivo* studies have validated Diosmin’s extensive range of biological capabilities which present antioxidative, antihyperglycemic, anti-inflammatory, antimutagenic, and antiulcer properties. We have presented this review work because of the greater biological properties and influences of Diosmin. We have provided a brief overview of Diosmin, its pharmacology, major biological properties, such as anti-cancer, anti-diabetic, antibacterial, anticardiovascular, liver protection, and neuroprotection, therapeutic approach, potential Diosmin targets, and pathways that are known to be associated with it.

## 1. Introduction

Diosmin is a flavonoid found in citrus fruits. Flavonoids are anti-inflammatory plant compounds that protect your body from free radicals and other unstable molecules. The most prevalent uses for Diosmin include hemorrhoids and leg sores caused by poor blood flow. It is also claimed to heal a variety of diseases, albeit there is no hard evidence to back up these claims [1,2,3]. Hesperidin is frequently used with Diosmin which is another plant chemical. Diosmin may work by reducing swelling and restoring normal vein function. It also appears to have antioxidative effects [1,4,5,6]. Diosmin was first found in 1925 in the wort plant and has since been used as a natural treatment for hemorrhoids, varicose veins, venous insufficiency, leg ulcers, and other circulatory issues [7]. It may help people with venous insufficiency, a condition in which blood flow is restricted, reduce inflammation, and restore normal blood flow [8,9,10,11].

**Figure 1:**
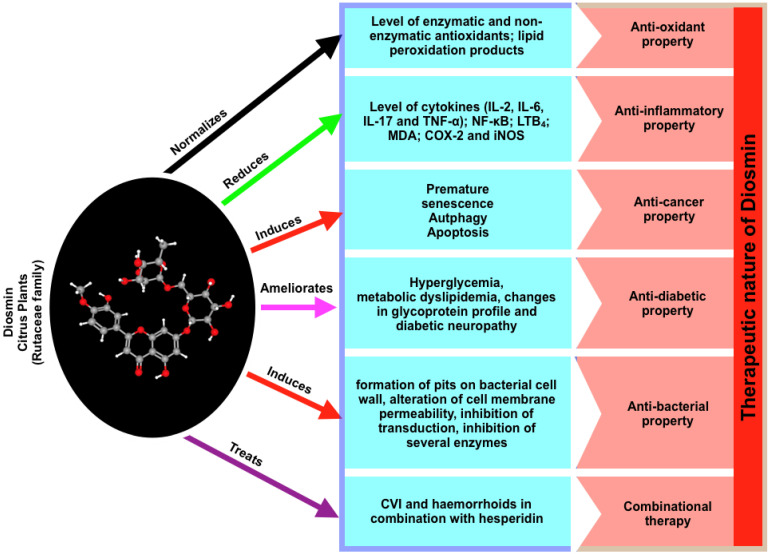
A summary of Diosmin, the associated diseases and potential properties in terms of disease protection.

Due to its beneficial effects in a number of major human organ systems, such as the cardiovascular system, Diosmin is now one of the most sought-after natural compounds in the treatment of a variety of human diseases, including chronic venous insufficiency (CVI), a progressive disorder affecting an increasing number of people (Figure 1). Diosmin’s effects in combination with other flavonoids, as well as its delivery routes, have also been investigated in details [4,12,13,14]. Diosmin was shown to have anti-oxidant characteristics, allowing it to successfully modulate the activities of a range of variables (including enzymes and biomarkers) connected to oxidative imbalance in a variety of illnesses. Induced apoptosis has been shown to have anti-cancer characteristics and activity in a variety of cell types. In MCF-7, MDA-MB-231, and SKBR-3 breast cancer cells, as well as DU145 prostate cancer cells, A431 skin cancer cells, and colon, oral, urinary bladder, and esophageal carcinogenesis, Diosmin has been found to trigger apoptosis [15,16,17,18]. Diosmin has also been found to have anti-diabetic properties. Diosmin has therapeutic effects in the event of diabetes and associated complications, such as neuropathy and dyslipidemia. Some evidence of a weak antimicrobial action was also found. A combination of Diosmin and hesperidin has been shown to be particularly effective in the treatment of chronic venous insufficiency and hemorrhoids. Diosmin is a great medical medication that can be used alone or in conjunction with other flavonoids [1,14,17].

Intestinal microflora enzymes rapidly hydrolyze Diosmin into its aglycone form, diosmetin, which is easily absorbed and dispersed throughout the body, according to previous pharmacokinetic studies. In addition, Diosmin is broken down into phenolic acids or glycine-conjugated derivatives, which are excreted in the urine. The presence of break down products such as alkyl-phenolic acids demonstrated that the flavonoids followed a similar metabolic path to other flavonoids [12,18,19,20].

Diosmin’s anti-oxidant properties were discovered to have beneficial effects in a number of illnesses, where it successfully reduced the activities of several components associated to oxidative imbalance, such as enzymes/biomarkers. Induced apoptosis has been shown to have anti-cancer characteristics and activity in a variety of cell types [7,11,21]. In cancer cell lines, such as MCF-7, MDA-MB-231, SKBR-3; DU145; A431; colon, oral, urinary bladder, and esophageal carcinogenesis, Diosmin promotes apoptosis. Diosmin has also been found to have anti-diabetic properties. Diosmin has therapeutic effects in the event of diabetes and associated complications, such as neuropathy and dyslipidemia. Some evidence of a weak antimicrobial action was also found. A combination of Diosmin and hesperidin has been shown to be particularly effective in the treatment of chronic venous insufficiency and hemorrhoids. Diosmin is a great medical medication that can be used alone or in conjunction with other flavonoids [12,18,19,20].

Oxidative stress has been linked to the development of various diseases, including myocardial ischemia, cerebral ischemia–reperfusion injury, diabetes, neuronal cell injury, hypoxia, and cancer [16,22]. In rat models, Diosmin was found to exhibit anti-oxidant effects and to stimulate human neutrophils. Because of its anti-oxidant properties, Diosmin has been shown to offer a variety of therapeutic effects for illnesses characterized by oxidative stress. Many diseases, including arthritis, allergies, asthma, autoimmune diseases, atherosclerosis, diabetes, and cancer, are caused by inflammation. Inflammatory indicators, or biomarkers with elevated levels, can be used to detect inflammation [18,23]. The most common inflammatory markers include immune system cells, such as neutrophils, basophils, eosinophils, platelets, macrophages, and others, cell surface receptors and adhesion molecules such as selectins (L-selectin, P-selectin, and E-selectin), and soluble mediators such as cytokines (IL-1, IL-2, IL-6, TNF-α, TGF-ß, and IFN-γ) (complement factors, C-reactive protein and the coagulation factor fibrinogen) [8,10,15,18].

Recently published studies have shown that Diosmin has dose-dependent pro-apoptotic effects on a range of animal cancers, including breast, prostate, colon, oral, and urinary bladder tumors. Diosmin has been reported to trigger premature senescence in a variety of cancer cell types [23,24]. Other breast cancer cell lines, including MDA-MB-231 and SKBR-3, responded to Diosmin, but MCF-7 was found to be the most responsive. At lower doses, Diosmin produced G2/M cell cycle arrest, elevated p53, p21, and p27 levels, increased SA—gal activity, oxidative stress, and DNA damage in MCF-7 cells, all of which are associated with ageing. Apoptosis can be triggered by increased levels of nitric oxide, total ROS, total superoxide, mitochondrial production, and protein carbonylation. In the treatment of diabetes and its complications, Diosmin has been proven to have therapeutic effects. Diabetes mellitus is a chronic disease characterized by abnormal glucose, protein, and lipid metabolism due to a lack of or reduced insulin action. Diosmin possesses anti-hyperglycemic effects, according to numerous studies [13,14,17,22,25].

Plant-based bioactive components that are effective against fungus, yeasts, and bacteria, as well as insects, nematodes, and other plants, are known as phytochemicals. They can inhibit the synthesis of peptidoglycans, damage microbial membrane structures, alter the hydrophobicity of bacterial membrane surfaces, and interfere with quorum sensing (QS). The antibacterial activities of Diosmin were previously investigated by synthesizing silver nanoparticles from it (AgNPs). Flavonoids-rich diets aid in the improvement of cardiovascular parameters. Anti-platelet activity has been demonstrated for Diosmin. Overall binding in the heparin binding region, which includes helix A, D, and N-terminal residues, is expected to improve with Diosmin sulfation, potentially leading to the development of new anti-thrombin candidates. Diosmin’s anti-thrombotic properties are confirmed by changes in the protein composition of rats with venous thrombosis. Diosmin may stimulate endothelial cell growth by targeting the centrosome-associated protein 350 (CEP350) [5,13,26,27].

Hypoxia, angiogenesis, inflammation, and intrapulmonary vasodilation characterize hepatopulmonary syndrome (HPS), a severe consequence of hepatic cirrhosis. TNF/VEGF, IGF-1/PI3K/AKT, and FGF-1/ANG-2 signaling pathways may be involved in HPS development and can be restored by Diosmin therapy in a chronic bile duct ligation (CBDL)-induced rat model. In the central nervous system, many flavone derivatives have been found as potential GABAA receptor ligands, where they interact with the benzodiazepine binding site to generate depressive effects. Flavonoid glycosides cannot directly regulate the activity of the GABAA receptor. Diosmin contains sedative and sleep-inducing characteristics that have nothing to do with GABAA receptor regulation. 6-C-glycoside-diosmetin has been shown to have memory-enhancing and anxiolytic-like effects by activating the GABAA receptor, which is controversial [7,12,23].

## 2. Pharmacology of Diosmin

Flavonoids are phytochemicals that have been proven to have medicinal properties [28,29,30]. Diosmin, a flavone glycoside, is also found in hesperidin [9,12]. Daflon is an oral phlebotonic flavonoid combination based on Diosmin and Hesperidin that is widely used to treat and protect blood vessel disorders. After oral administration, Diosmin is converted to diosmetin, which is subsequently absorbed and esterified into glucuronide conjugates that are excreted in the urine [11,23,24,31]. Diosmin’s pharmacological properties have been investigated in a number of in vitro and in vivo studies, and it has been found to have anti-inflammatory, antioxidant, antidiabetic, antibacterial, antihyperlipidemic, and antifibrotic properties in a variety of disease models, as described in the previous section. In a number of clinical studies, Diosmin has also been proven to have a number of positive properties. According to toxicological investigations, Diosmin has a favorable safety profile. As a result, Diosmin has the potential to be an effective and safe treatment for a wide range of conditions. Diosmin, on the other hand, inhibits a variety of metabolic enzymes, encouraging clinical trials to look into its potential therapeutic efficacy and safety in a variety of illnesses while taking into consideration potential interactions. Diosmin is a venoactive drug that works on blood vessels in a variety of ways to improve circulation. It improves lymphatic drainage and microcirculation, as well as venous tone and suppleness. Diosmin is often used to improve vascular health in people with chronic venous disease for these reasons, and it has been demonstrated to improve quality of life. Diosmin also has antioxidant characteristics and scavenges oxygen free radicals, lowering oxidative stress levels that can be detected by biomarkers such as prostaglandin isoprostane precursors [2,11,12,31,32].

## 3. Critical Properties of Diosmin

### 3.1. Anti-Oxidant Property

Oxidative stress has been linked to the development of various diseases, including myocardial ischemia, cerebral ischemia–reperfusion injury, diabetes, neuronal cell injury, hypoxia, and cancer. In rat models, Diosmin was found to exhibit anti-oxidant effects and to stimulate human neutrophils. Because of its anti-oxidant properties, Diosmin has been shown to offer a variety of therapeutic effects for illnesses characterized by oxidative stress [22,33,34,35,36,37,38,39,40]. Diosmin was found to have significant anti-diabetic effects in diabetic rats fed streptozotocin nicotinamide (STZ-NA). When diabetic control rats were compared to normal control rats, anti-oxidant enzyme activities, such as glutathione-S-transferase (GST), glutathione peroxidase (GPx), superoxide dismutase (SOD), and catalase (CAT), as well as levels of low molecular weight antioxidants, such as vitamin C, vitamin E, and reduced glutathione (GSH), were found to be low, whereas lipid peroxidation Hyperglycemia with a significant drop in plasma insulin levels was also seen [7,11,27].

Oral administration of Diosmin enhanced the glycemic and anti-oxidant status of diabetic rats, according to previous studies. Diosmin treatment was also found to reduce lipid peroxidation [21,22,41]. Due to its anti-oxidant properties, Diosmin functions as an anti-hypertensive drug in deoxycorticosterone acetate (DOCA)-salt-induced hypertensive rats. Hypertension is characterized by an excessive production of reactive oxygen species. In DOCA-salt-treated rats, non-enzymatic and enzymatic antioxidants were found to be lower, while lipid peroxidation products (thiobarbituric acid reactive substances, lipid hydroperoxides, and conjugated dienes) were found to be significantly higher in blood plasma and tissues, such as the liver, kidney, heart, and aorta. Antioxidant levels were restored to near-normal levels, and lipid peroxidation products were reduced as a result of the Diosmin therapy. These findings were confirmed by histopathological examinations of the kidney and heart [1,11,31,42] Diosmin was found to have a hepatoprotective effect against ferrous sulfate-induced liver damage in adult male albino rats. Too much iron causes oxidative stress, reactive oxygen species (ROS), lipid peroxidation, inflammation, and tissue necrosis. Elevated ALT, AST, ALP, GGT, LDH activity, and bilirubin levels indicate hepatocyte membrane damage [21]. After Diosmin treatment, these values were considerably normalized. Because it maintains membrane integrity, decreases oxidative stress, and aids in the correction of dyslipidemia, Diosmin is a good hepatoprotective drug. The antioxidant and anti-inflammatory properties of Diosmin are primarily responsible for its hepatoprotective effects [11]. According to Senthamizhselvan et al., pre-treatment with Diosmin reduces oxidative stress in the rat heart after ischemia/reperfusion [43]. Ischemia–reperfusion injury occurs when ischemic tissue is reperfused, causing oxidative stress and cellular damage. The activities of enzyme antioxidants (SOD, CAT, and GPx) and GSH levels declined when hearts of control rats (no Diosmin pre-treatment) were subjected to an ischemia/reperfusion regimen, although the levels of lipid peroxidation products rose [44,45]. After 7 days of oral therapy with Diosmin (50 and 100 mg/kg), the activities of enzymatic antioxidants and GSH levels were found to be increased, while the levels of lipid peroxidation products were found to be reduced [6,43]. Ischemia–reperfusion injury produces edema and tissue destruction in the retina, resulting in vision loss. Diosmin was discovered to help male Wistar rats with retinal edema by maintaining the blood-retinal barrier and lowering vascular permeability. Diosmin’s ability to change the VEGF/PEDF ratio could explain its protective properties. The levels of malondialdehyde (MDA) and the activity of total-superoxide dismutase (T-SOD), glutathione peroxidase (GSH-Px), and catalase (CAT) in retinal tissues that had been altered by ischemia–reperfusion injury, recovered to normal after Diosmin administration (Tong et al., 2013). Diosmin protects male CD-1 mice from brain ischemia–reperfusion injury by stimulating the JAK2/STAT3 signaling pathway [3,13]. In human polymorphonuclear neutrophils stimulated in vitro by phorbol myristate acetate, Cypriani et al. discovered that S5682 (Daflon 500 mg), a purified flavonoid fraction including Diosmin and hesperidin in a 9:1 ratio, has anti-oxidative effects (Cypriani et al., 1993). Diosmin’s free-radical scavenging activity is one of the reasons for its protection against myocardial infarction [42,46].

### 3.2. Anti-Inflammatory Property

Many diseases, including arthritis, allergies, asthma, autoimmune diseases, atherosclerosis, diabetes, and cancer, are caused by inflammation. Inflammation can be detected by measuring the levels of certain biomarkers known as inflammatory markers. Immune cells, such as neutrophils, basophils, eosinophils, platelets, macrophages, and others, cell surface receptors and adhesion molecules such as selectins (L-selectin, P-selectin, and E-selectin), and soluble mediators, such as cytokines (IL-1, IL-2, IL-6, TNF-α, TGF-ß, and IFN-γ), chemokines, and NF-kB (complement factors, C-reactive protein and the coagulation factor fibrinogen) [8,9,10,15,18].

Diosmin has been shown in multiple studies to lower these markers due to its anti-inflammatory effects. In lung damage produced by lipopolysaccharide (LPS) therapy, pro-inflammatory cytokines (IL-2, IL-6, IL-17, and TNF-α) and NF-kB were shown to be increased. After a few days of Diosmin pre-treatment, the levels of these indicators were dramatically lowered. In this study, male adult Balb/c mice were employed (Imam et al., 2015). In rat colitis caused by trinitrobenzenesulfonic acid, Diosmin was found to decrease the production of LTB4 (eicosanoid) and colonic MDA (TNBS). In the TNBS-treated colon, LTB4 improves neutrophil chemotaxis, adhesion, and degranulation, whereas MDA is a lipid peroxidation product (Crespo et al., 1999). According to Tahir et al., Diosmin reduces COX-2 and iNOS levels in chemically induced hepatocarcinogenesis (inflammatory markers). In female Wistar rats, diethylnitrosamine (DEN) was utilized to induce hepatocarcinogenesis, while 2-acetylaminofluorene was employed to promote it (2-AAF). In a long-term experiment, Diosmin was given orally at doses of 10 and 20 mg/kg b.wt for 9 weeks (Tahir et al., 2013). Diosmin treatment was found to alter the levels of tumor necrosis factor (TNF-α) and cyclooxygenase-2 in acetic acid-induced ulcerative colitis (COX-II). TNF-α and COX-II levels were lowered in a dose-dependent manner in Diosmin-treated rats [10].

### 3.3. Anti-Cancer Property

**Figure 2:**
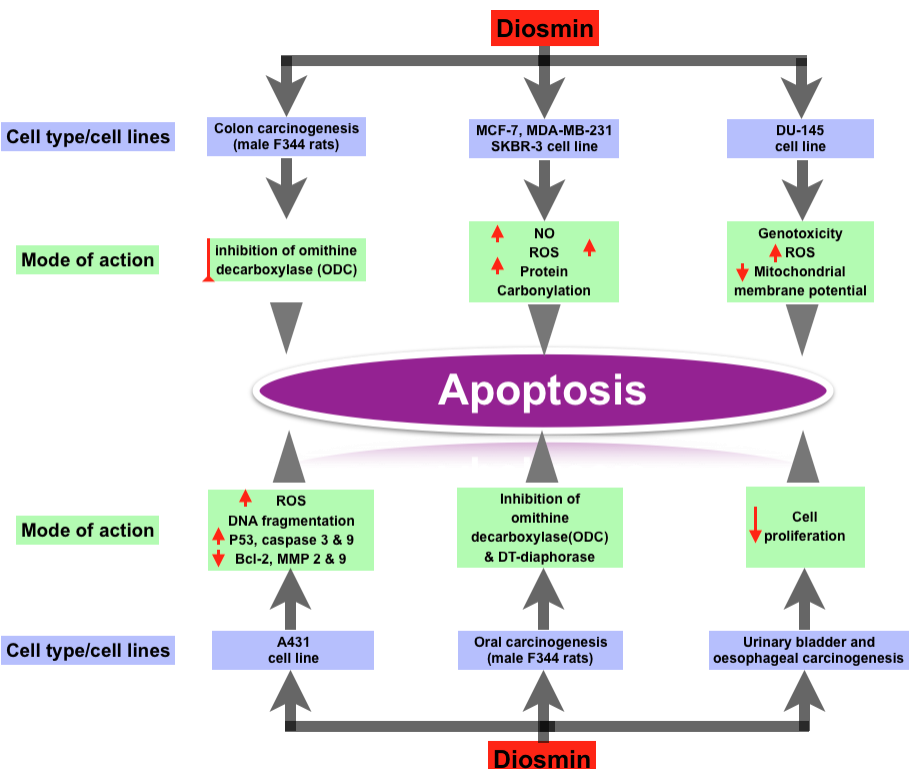
Effect of Diosmin on different types of cancer cell lines and their mode of actions.

Recent study has discovered that Diosmin has dose-dependent pro-apoptotic effects on a range of animal cancers, including breast, prostate, colon, oral, and urinary bladder tumors. Diosmin causes premature senescence and apoptosis in MCF-7 cells at various doses. Other breast cancer cell lines, including MDA-MB-231 and SKBR-3, responded to Diosmin, but MCF-7 was found to be the most responsive. At lower doses, Diosmin produced G2/M cell cycle arrest, elevated p53, p21, and p27 levels, increased SA—gal activity, oxidative stress, and DNA damage in MCF-7 cells, all of which are associated with ageing. Apoptosis can be triggered by increased levels of nitric oxide, total ROS, total superoxide, mitochondrial production, and protein carbonylation. In research on the androgen-independent prostate cancer cell line DU145, Diosmin’s pro-apoptotic activity was confirmed. The genome and cytotoxicity of three flavonoid glycosides (Diosmin, naringin, and hesperidin) were studied in the DU145 prostate cancer cell line. The most genotoxicity was caused by Diosmin. In DU145 cells, these flavonoids caused oxidative stress or intracellular redox disequilibrium, leading to changes in mitochondrial membrane potential and apoptotic cell death. Diosmin dramatically boosted overall ROS production. Diosmin therapy also increased the amount of double stranded breaks in DNA and the formation of micronuclei (genotoxicity) [16]. Diosmin has anti-proliferative effect in human colon cancer cell lines, according to Kuntz et al. [29]. Tanaka et al. discovered a chemopreventive effect of Diosmin on colon carcinogenesis produced by azoxymethane in male F344 rats [19,47]. The treatment of Diosmin orally reduced colon carcinogenesis, as evidenced by lower rates of colon cancers. They hypothesized that inhibiting ornithine decarboxylase (ODC), a rate-limiting enzyme in polyamine production, was responsible for the decrease in colonic cancers [47]. Cell apoptosis is produced by DNA damage when ODC is inhibited [48]. When exposed to carcinogens, ODC levels have been reported to rise in numerous tissues. ODC activity was likewise elevated in the colonic mucosa of azoxymethane-treated rats. Diosmin has a dose-dependent cytotoxic capability for A431 skin cancer cells, according to Buddhan et al. It inhibits the invasive capacity of A431 cells by inducing apoptosis through a ROS-mediated mechanism [21,27]. DNA fragmentation, overexpression of p53, caspase 3 and caspase 9 genes, and downregulation of Bcl-2, matrix metalloproteinases-2 and 9 genes were all observed in A431 cells after Diosmin treatment. Diosmin’s IC50 value was discovered to be 45 g/mL, at which point it produced significant ROS [49]. Diosmin was found to prevent oral carcinogenesis produced by 4-nitroquinoline 1-oxide in male F344 rats (4-NQO). They theorized that Diosmin had anti-cancer capabilities via a number of mechanisms. One of these mechanisms was the inhibition of ornithine decarboxylase (ODC). Agents that inhibit ODC activity have been shown to be effective in slowing tumor growth. Another hypothesized mechanism is the inhibition of DT-diaphorase activity, which is required for 4-NQO to have its carcinogenic effect [19,47]. Inhibiting oral carcinogenesis, Diosmin is more efficient than diosmentin (the aglycone version of Diosmin) [50]. Oral treatment of 1000 ppm Diosmin suppressed urinary-bladder carcinogenesis induced by N-butyl-N-(4-hydroxybutyl) nitrosamine in male ICR mice. These findings were supported by a count of silver-stained nucleolar-organizer-region-associated proteins (AgNORs) and a 5-bromodeoxyuridine (BUdR)-labeling index. Diosmin’s anti-carcinogenic effects may be due to a decrease in cell proliferation [13,51]. In male Wistar rats, Diosmin had a similar effect on esophageal carcinogenesis induced with N-methyl-N-amylnitrosamine (MNAN). Diosmin’s chemopreventive effects on several cancer kinds or cell lines are summarized in Figure 2. 

Diosmin has also been shown to have anti-metastatic properties in metastatic lung melanoma (B16F10) [52]. Diosmin lowers the number of metastatic nodules, implant percentage, and invasion index in both micro- and macroscopic investigations. In the treatment of metastatic pulmonary melanoma, Diosmin acts in tandem with IFN-γ [53,54]. By disrupting the PI3K–Akt–MDM2 signaling pathway, Diosmin decreased HA22T cell growth (human hepatocellular cancer) in nude mice models and triggered G2/M cell cycle arrest through p53 activation [24,55].

### 3.4. Anti-Diabetic Property

In the treatment of diabetes and its complications, Diosmin has been proven to have therapeutic effects. Diabetes mellitus is a chronic disease characterized by abnormal glucose, protein, and lipid metabolism due to a lack of or decreased insulin activity. Diosmin possesses anti-hyperglycemic effects, according to numerous studies. According to Pari et al., Diosmin (in various doses) taken orally for 45 days can improve glycemic control. In the study, male albino Wistar strain rats were given streptozotocin-nicotinamide to develop diabetes (STZ-NA) [22,41]. The effect of Diosmin on plasma glucose levels was shown to be dose-dependent. In addition, oral Diosmin (100 mg/kg b.w.) treatment decreased glycosylated hemoglobin while raising hemoglobin and plasma insulin. Hexokinase and glucose-6-phosphate dehydrogenase, two essential liver enzymes, were also suppressed. In addition, there was a rise in body weight [22,41].

Diosmin has also been linked to improved lipid metabolism in people with diabetes. Hypercholesterolemia, lipid buildup in hepatic organs, and alterations in plasma lipid and lipoprotein profiles are all symptoms of metabolic dyslipidemia in people with type-2 diabetes [56,57]. Plasma lipids, tissue lipids (cholesterol, TGs, FFAs, and PLs), and plasma lipoproteins can all be efficiently normalized with Diosmin therapy (LDL, VLDL) [22,41]. Diosmin also corrects changes in glycoprotein profile caused by type-2 diabetes. When glucose is utilized by insulin-independent processes in diabetic people, glycoproteins build up. In STZ-NA-induced diabetic rats, the level of plasma glycoproteins increased dramatically. In diabetic rats’ liver and kidneys, hexose, hexosamine, and fucose levels were much greater, but sialic acid levels were significantly lower. Diosmin, when taken orally, has been shown to counteract these changes in glycoprotein profile [22,41].

Neuropathy is one of the most common diabetic consequences, affecting more than half of those who have the illness. Chronic untreated and uncontrolled hyperglycemia causes pain, tingling, and numbness in the periphery, as well as delayed nerve conduction. Hyperglycemia-induced oxidative stress results in the accumulation of polyols and advanced glycation end products, as well as impairment of (Na+/K+)-ATPase activity and endothelial function. Apoptosis is the death of neurons as a result of oxidative stress. Jain et al. employed streptozotocin and a high-fat diet to induce type-2 diabetes in male Sprague-Dawley rats to assess the effect of Diosmin on diabetic neuropathy. In rats, four weeks of treatment with Diosmin (50 and 100 mg/kg, p.o.) reduced the development of early diabetic neuropathy. It reduced oxidative stress by restoring GSH, NO, and SOD activity that had been changed [27,31,42,58]. Diosmin treatment resulted in considerable NF-kB normalization in alloxan-induced diabetic Wistar mice. NF-kB is important in the pathogenesis of diabetic neuropathy and other inflammatory illnesses [8]. In male Swiss mice, Diosmin was discovered to alleviate neuropathic pain produced by chronic constriction injury (CCI). Diosmin (1 or 10 mg/kg) was given intraperitoneally to reduce CCI-induced mechanical and thermal hyperalgesia. The role of the NO/cGMP/PKG/KATP channel signaling pathway was verified using inhibitors, such as L-NAME (an inhibitor of NOS), ODQ (an inhibitor of soluble guanylate cyclase), KT5823 (an inhibitor of PKG), or glibenclamide (an ATP-sensitive K+ channel blocker). Diosmin treatment also inhibited the production of cytokines (IL-1 and IL-33/St2) and reduced the activation of glial cells [5,31,59].

### 3.5. Anti-Bacterial Property

In bacteria, the majority of medications have developed resistance. Plants are being researched in the hope of discovering new and effective antibacterial agents. Plant-based bioactive components that are effective against fungus, yeasts, and bacteria, as well as insects, nematodes, and other plants, are known as phytochemicals. They can inhibit the synthesis of peptidoglycans, damage microbial membrane structures, alter the hydrophobicity of bacterial membrane surfaces, and interfere with quorum sensing (QS). By synthesizing silver nanoparticles of Diosmin, it has been shown the antibacterial properties (AgNPs) where they have tested its antimicrobial activity against Escherichia coli, Pseudomonas putida, and Staphylococcus aureus using the disc diffusion method. Diosmin produced hexagonal AgNPs that were slightly antimicrobial and had a size of roughly 5–40 nm. Diosmin inhibited E. coli, P. putida, and S. aureus with zones of inhibition of 6, 6, and 7 mm, respectively. Pits on the bacterial cell wall, changes in cell membrane permeability, obstruction of transduction, suppression of respiratory enzyme function due to free radical generation, and inactivation of various thiol-containing enzymes have all been proposed as antibacterial mechanisms [13,17,51].

Diosmin with amoxicillin-clavulanic acid (AMC) has been found to have mycobactericidal efficacy against Mycobacterium marinum. After treatment with a combination of AMC and Diosmin, the survival of M. marinum-infected Drosophila melanogaster fly models improved by 60%, providing in vitro proof. Its antibacterial activity was also proven against Mtb H37Ra and an MDR clinical isolate. The AMC-Diosmin combination was discovered to target L, D-transpeptidase (LDT) enzymes involved in Mtb cell wall synthesis, resulting in cellular leakage in *M. marinum* cells [14].

### 3.6. Cardiovascular Protection

Diets high in flavonoids help promote cardiovascular health. Diosmin has been shown to have anti-platelet action. Sulfation of Diosmin will very certainly improve overall binding at the heparin binding site, which contains helix A, D, and N-terminal residues, resulting in the development of novel anti-thrombin candidates. Diosmin’s anti-thrombotic properties are confirmed by changes in the protein composition of rats with venous thrombosis. Diosmin, which targets centrosome-associated protein 350, may increase endothelial cell proliferation (CEP350). In a rat model produced by the nitric oxide production inhibitor L-NAME, Diosmin’s antihypertensive effects are demonstrated [4,7,11,60,61,62,63,64]. Diosmin appears to protect rats from myocardial infarctions, hyaline arteriopathy, and fibrinoid necrosis caused by L-NAME. The elimination of superoxide anions by Diosmin could be the underlying mechanism for its anti-hypertensive actions. Diosmin has been shown to lower serum cardiac marker enzyme production, reduce plasma lipid peroxidation, and restrict lipid metabolism abnormalities in isoproterenol-induced myocardial-infarcted rats, resulting in anti-hyperlipidemia and cardio-protection. Isoproterenol has been reported to raise cardiac diagnostic markers, heart mitochondrial lipid peroxidation, and calcium ions while lowering anti-oxidant enzyme expression. Diosmin has been shown to be useful in preventing these traits. Left ventricular hypertrophy (LVH), ATPase dysfunction, and electrolyte imbalance all play a role in the etiology of isoproterenol-induced myocardial infarction. Pretreatment with Diosmin may attenuate the degenerative effects of isoproterenol in rats [4,5,7,11,31,65].

Reperfusion of ischemic tissues typically causes the generation of free radicals. Heart function recovery, anti-oxidant enzyme expression, and lipid peroxidation are all protected by Diosmin. In the presence of reperfusion stress, Diosmin successfully preserves TCA cycle enzyme activity. Diosmin reduces metabolic syndrome-related cardiovascular issues in rats, as demonstrated by improvements in systolic and diastolic blood pressure (BP) and ECG parameters. The anti-oxidant and anti-inflammatory effects of Diosmin in rats could explain these findings. Diosmetin protects mice from damage caused by ISO by upregulating AKT and NRF2 signaling while suppressing the NF-kB pathway. In venous smooth muscle action, Ca^2+^ is a key mediator. The contraction of the inferior vena cava (IVC) in normal Krebs and Ca^2+^-free Krebs has been shown to be unaffected by Diosmin. Diosmin, on the other hand, enhances the contractile response generated by KCl [23,24].

Sclerotherapy is a telangiectasia and varicose veins treatment that might cause irreversible endothelial damage. An exaggerated inflammatory response is usually induced during the sclerotherapy technique. MPFF has been shown to be useful in lowering inflammatory stress during sclerotherapy. By increasing venular diameter, preserving functional capillary density, reducing the number of leaky sites, and binding leukocytes, MPFF reduces post-sclerotherapy inflammation in a microvascular network. MPFF has been shown to lower the production of metalloproteinase-2 (MMP-2) and MMP-9 in rats while boosting MDA, which helps with varicose vein therapy. Linfadren, a combination of Diosmin, coumarin, and arbutin, has been shown in a randomized controlled trial to treat chronic hand edema in patients with post-trauma/surgery. Linfadren has been demonstrated to assist patients with breast cancer-related lymphedema when used in conjunction with extensive decongestive therapy [1,21,42,66].

Primary reflux from primary valve incompetence and venous thrombosis induces chronic venous illness, which includes pain, edema, skin damage, and ulceration. Two possible explanations are venous hypertension and microcirculation problems. Inflammation is both the beginning and the end of primary valve incompetence, as well as venous hypertension. Daflon 500 mg relieves clinical symptoms by reducing inflammatory reactions not only in the microcirculation but also in the vein wall and valve cusps. According to randomized trials, Daflon 500 mg for 60 days of therapy is also effective in elastic compression and speeding up the healing process in venous ulcers. A meta-analysis of Daflon 500 mg’s effects on venous leg ulcers found that it can be a valuable addition to standard therapy in big and long-standing ulcers. In randomized, double-blind, controlled trials, the therapeutic efficacy of Daflon 500 mg on chronic venous disease symptoms and edema was also studied. Diosmin may significantly reduce angiogenesis and inflammation, as evidenced by downregulation of TNF-α, IL-6, VEGF-C, VEGF-A, and FGF2 expression and overexpression of angiostatin expression in individuals with chronic venous issues. After six months of Daflon treatment, however, air plethysmography revealed no changes in venous hemodynamics. By magnifying adrenergic impact on microcirculation, Diosmin may cause adverse effects by raising creatine phosphokinase and serum lactic dehydrogenase levels [4,67,68,69].

Finally, Diosmin may protect against myocardial infarctions, hyaline arteriopathy, and fibrinoid necrosis caused by L-NAME by reducing serum cardiac marker enzyme production, plasma lipid peroxidation, and lipid metabolism alterations. By correcting isoproterenol-induced LVH, ATPase failure, and electrolyte imbalance, it improves metabolic syndrome-related cardiovascular issues. MPFF decreases MMP-2 and MMP-9 expression while increasing MDA, improving venular diameter, maintaining functional capillary density, reducing leaky sites, and sticking leukocytes. Inflammatory responses in the microcirculation, vein wall, and valve cusps are reduced with Daflon 500 mg. Daflon 500 mg has also been shown to be effective in the treatment of chronic venous disease symptoms and edema. TNF, IL-6, VEGF-C, VEGF-A, and FGF2 expression were all downregulated, whilst angiostatin expression was upregulated, showing that Diosmin reduces angiogenesis and inflammation in chronic venous disease patients. As evidenced by increases in SOD, CAT, GSH, and NO, as well as a decrease in MDA, Diosmin maintains redox equilibrium and downregulates the NF-B signaling pathway. Diosmin also lowers kidney weight, pH, total protein, calcium, and phosphorus in the urine, as well as potassium, sodium, magnesium, creatinine, and uric acid in the blood.

### 3.7. Liver Protection

Hypoxia, angiogenesis, inflammation, and intrapulmonary vasodilation characterize hepatopulmonary syndrome (HPS), a severe consequence of hepatic cirrhosis. TNF/VEGF, IGF-1/PI3K/AKT, and FGF-1/ANG-2 signaling pathways may be involved in HPS development and can be restored by Diosmin therapy in a chronic bile duct ligation (CBDL)-induced rat model. According to another study, Diosmin decreases BDL-induced liver abnormalities through modifying the Keap-1/NRF2 and p38-MAPK/NF-B/iNOS signaling pathways. Diosmin, pentoxifylline, and their combination have been shown to reduce BDL-induced liver cirrhosis via modulating the Keap-1/Nrf-2/GSH and NF-kB/p65/p38-MAPK signaling pathways. Diosmin also stimulates the production of cytoglobin, which contributes to the compound’s anti-oxidant, anti-inflammatory, and anti-fibrotic properties. Diosmin promotes the NRF2/Keap-1 pathway while suppressing the ROS-induced p38 MAPK pathways and activating the eNOS gene. By binding to the ARE sequence and upregulating the production of target genes, such as HO-1 and SOD, active NRF2 may enter the nucleus to promote transcriptional activity. The activity of the NF-kB, p53, and iNOS signaling pathways may be boosted when the p38 MAPK is triggered [6,7,13,27,41].

### 3.8. Neuroprotection

Diosmin performs a variety of actions that are linked to neuroprotection. Diosmin is a sedative that induces sleep and decreases the expression of IL-1, TNF, and IL-33/St2, as well as activating glial cells. It lowers mechanical and thermal hyperalgesia by activating D2, GABAA, and opioid receptors but not 5-HT1A receptors, reduces neuropathic pain by activating the NO/cGMP/PKG/KATP pathway, and suppresses IL-1, TNF-a, and other inflammatory mediators. Diosmin has been associated to thermal hyperalgesia, cold allodynia, and walking dysfunctions, as well as oxidative stress. It also helps with scopolamine-induced neural plasticity disruption and cognitive deficits by inhibiting TNF expression. Diosmin has an IC50 of 12.24 0.54 g mL and interacts with AChE enzyme via Tyr72, Tyr124, Trp286, Phe295, and Tyr341. Many flavone derivatives have been discovered as possible GABAA receptor ligands in the central nervous system, where they interact with the benzodiazepine binding site to produce depressive effects. The activity of the GABAA receptor cannot be controlled directly by flavonoid glycosides. Diosmin has sedative and sleep-inducing properties that are unrelated to GABAA receptor modulation. 6-C-glycoside-diosmetin activation of the GABAA receptor has been shown to have memory-enhancing and anxiolytic-like effects, which are controversial. As a result, more research into flavone derivatives’ interactions with the GABAA receptor is required. A range of harmful compounds in the peripheral and central nerve systems induces neuropathic pain. Diosmin coupled with hesperidin effectively reduces mechanical or thermal hyperalgesia in the chronic constriction injury (CCI) rat model. D2, GABAA, and opioid receptor antagonists may prevent these effects, while the 5-HT1A receptor inhibitor does not. In a CCI rat model, Diosmin alone has been shown to have anti-hyperalgesic effects via D2 and opioid receptors, as well as a reduction in pro-inflammatory cytokine (IL-1, TNF, and IL-6) expression [5,7].

Diosmin was found to reduce CCI-induced neuropathic pain by activating the NO/cGMP/PKG/KATP channel signaling pathway, lowering spinal cord cytokine (IL-1, TNF, and IL-33/St2), and activating glial cells in a separate investigation. Thermal hyperalgesia, cold allodynia, and walking dysfunctions, as well as oxidative stress, may be caused by STZ-induced diabetes in rats. The diabetes abnormalities in the brain system can be successfully corrected with Diosmin. Furthermore, diosmetin inhibits nociception in mice via antagonizing the transient receptor potential vanilloid 1 (TRPV1) receptor [12].

Misfolded proteins have been linked to neurodegenerative disorders. Diosmin was discovered to bind to hen egg white lysozyme (HEWL) in a sheet-shape, in an in vitro research, minimizing HEWL aggregation. These may play a role in treating amyloid-related illnesses. The synthesis and aggregation of amyloid-(A) peptide aids the degenerative course of Alzheimer’s disease (AD). Inhibiting GSK-3 using Diosmin and its aglycone derivative diosmetin has been demonstrated to reduce brain soluble A and A oligomer formation, as well as tau hyperphosphorylation, and improve cognitive impairment in rats. Diosmin may aid to ameliorate scopolamine-induced synaptic plasticity disruption and cognitive impairments by reducing the expression of the pro-inflammatory cytokine TNF in the rat hippocampus. Traumatic brain injury (TBI) can cause mental and cognitive disability. Diosmin inhibits the pro-inflammatory cytokine TNF-α, protecting against TBI-induced declines in neurological scores, memory, and long-term potentiation (Yizhou Zheng, 2020a).

Diosmin appears to protect PC12 cells from LPS-induced TNF production and apoptosis, as seen by decreased DNA fragmentation, decreased Bad and caspase-3 expression, and enhanced Bcl-2 expression. Diosmin interacts with critical residues Tyr72, Tyr124, Trp286, Phe295, and Tyr341 in the acetylcholinesterase (AChE) enzyme and has an IC50 value of 12.24 0.54 g mL^−1^. This has a similar binding orientation to Donepezil. Diosmin’s inhibitory impact on the AChE enzyme has been confirmed in silico and in vitro experiments. In an ELISA experiment using the Ellman method, Diosmin had no effect on the activity of AChE and butyrylcholinesterase (BChE).

### 3.9. Additional Roles

During ischemia–reperfusion injury, Diosmin protects against retinal edema by maintaining tight junction integrity and lowering capillary permeability [37,41,56]. With an IC50 value of 24 M, Diosmin suppresses AR activity. Diosmin reduces phosphorylation levels of JNK and p38-MAPK, reducing oxidative stress and apoptosis. Diosmin mediates fast current low-voltage-activated K+ channels, voltage-independent K+ channels, and the nitric oxide pathway, acting as a myorelaxant. Diosmin’s anti-oxidative, anti-inflammatory, and anti-apoptotic activities reduce MTX-induced histological changes and restore tissue architecture. By enhancing anti-oxidant enzyme expression and lowering inflammatory cytokine production, Diosmin protects against alcohol-induced abnormalities. Diosmin protects against changes in antioxidant expression, body and organ weight, and histopathology caused by cadmium.

## 4. Potential Diosmin Target Proteins and the Pathways

**Figure 3:**
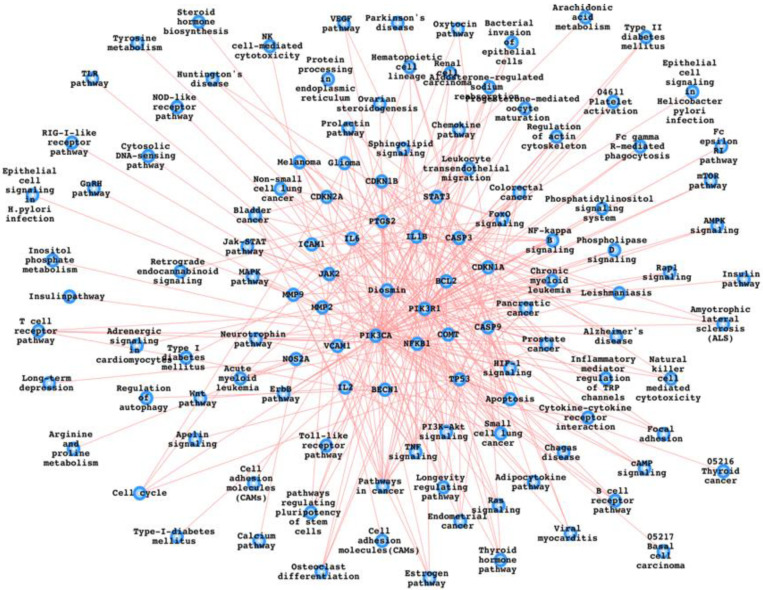
Diosmin-associated proteins and the biological pathways. We have used FunCoup database for PPI (protein–protein interactions) and integrated it with the KEGG pathways list, and the same has been mentioned in the main text also and the same has been done for Table 1 [72,73,74,75,76,77,78,79].

This article has gone over the proteins and pathways that have been identified as possible Diosmin targets. GSH and CAT are increased, while ROS, MDA, and lipid peroxidation are decreased. Diosmin is known to reduce the inflammatory proteins NF-kB, TNF-α, IL-1B, COX2, iNOS, and MAPKs. Diosmin controls the up (B-endorphin and glucose uptake) and down (gluconeogenesis and insulin resistance) of many diabetes-related pathways/pathway components [6,13,14,21]. Diosmin has been demonstrated to reduce proliferation, viability, and activate autophagy, all of which are known to play a role in various types of cancers. Platelet activity suppression, blood pressure, reperfusion stress, MMP-2/9, angiogenesis, and diagnostic markers are only a few of the cardiovascular parameters that Diosmin influences. Other pathways and their components linked to liver protection, neuroprotection, and antimicrobials include Keap-1, NRF2, p38-MAPK, iNOS, I/R-induced damage, EOF, RN4220, pUL5054, Ltd.Mt1, and Ltd.Mt2 [12,13,17,70,71]. Furthermore, we have mapped out the Diosmin target proteins and the respective pathways associated with them as shown in Figure 3. Furthermore, we have also presented a list of pathways which might be affected as a result of Diosmin intake (Table 1). 

To prepare this pathways list, we have first used SwissTargetPrediction (http://www.swisstargetprediction.ch/predict.php) (accessed on 21 December 2021) to generate the proteins which could bind with Diosmin and then mapped out the KEGG pathways from the KEGG database (https://www.genome.jp/kegg/pathway.html) (accessed on 21 December 2021) for these Diosmin interactors (proteins). Table 1 contains the overall pathways and the respective proteins while similar information is presented in Figure 3 as a network of Diosmin, its interactors, and the pathways.

## 5. Combinational Therapy, Side Effects, and the Administrative Route

Diosmin is a venoactive drug administered orally for the treatment of chronic venous insufficiency (CVI). In CVI, veins have trouble sending blood from limbs back to the heart as a result of which blood gets pooled in the veins of legs. Reflux of the venous valves is the most common cause of CVI [80,81,82]. Edema and symptoms of chronic venous disease (CVD), particularly so-called venous pain, are treated with venoactive medicines (VAD). The usefulness of VAD is contested on a regular basis, despite the fact that it is well documented. Our goal was to gather all randomized controlled trials (RCTs) and meta-analyses devoted to VAD and symptoms in CVD, present them to a panel of international CVD experts, and have them vote by secret ballot on the level of efficacy of each drug, using EBM (Evidence-Based Medicine) rules and critical analysis [82,83,84]. Diosmin in combination with hesperidin (Daflon 500 mg) has been found more effective than Diosmin alone on venous symptoms [4,82]. The most common symptoms of CVI include leg ache, sensation of heaviness or tension, nocturnal cramps, sensation of swelling, restless legs, and itching. Burning, heaviness, weakness, and functional pain are reduced after two months of Daflon treatment. It also enhances the skin microcirculation’s blood velocity. Daflon is not considered a cure for CVI because it does not address the underlying cause of the condition; rather, it is used to alleviate the symptoms of the disease. Diosmin and Micronized Purified Flavonoid Fraction (MPFF) have been found to affect venous tone, lymphatic drainage, and microcirculation in CVI patients. They improve venous tone by blocking COMT (catechol-O-methyltransferase) from breaking down norepinephrine (noradrenaline) and so prolonging noradrenergic action. They enhance the number of functional lymphatics, lymphatic flow, capillary hematocrit, and red cell velocity while decreasing lymphatic channel width and intra-lymphatic pressure. They also prevent leukocyte adherence, intra-tissue movement, and the release of leukocyte (L-selectin) and endothelial (ICAM-1, VCAM-1) adhesion molecules, which protect microvascular permeability (Inflammatory mediators) [21,66].

Daflon treatment for four weeks (four tablets per day, in two divided doses) has also shown improvement in the symptoms associated with hemorrhoids (pain, heaviness, bleeding, pruritus and anal discharge) [85,86,87]. However, purified Diosmin has also been shown to reduce pain and bleeding. Daflon has also been reported to exhibit anti-oxidative property. Combination of Diosmin with amoxicillin-clavulanic acid (AMC) has been found to possess mycobactericidal activity against *Mycobacterium Marinum* [14,20,88].

## 6. Future Perspectives

Diosmin is an anti-oxidant, anti-cancer, anti-diabetic, and mild anti-bacterial flavone glycoside derived from citrus trees. Because of its characteristics, it is a good therapeutic treatment for a variety of disorders. It reduces oxidative stress by altering the activity of particular enzymes and promotes apoptosis in a variety of cancer cell lines via several ways. Its anti-inflammatory properties are due to its ability to lower the levels of numerous inflammation markers. It also helps alleviate the consequences of diabetes, such as neuropathy and dyslipidemia. It has been found to be quite useful in the treatment of chronic venous insufficiency and hemorrhoids when combined with other flavonoids, particularly hesperidin. Treatment with Diosmin appears to be promising in the treatment of many malignancies, diabetes, and disorders linked to oxidative stress and inflammation. Diosmin is an anti-hyperglycemic drug that also helps with the issues that come with it. Its ability to modulate the VEGF/PEDF ratio could be investigated further to determine if it is a pro- or anti-angiogenic factor. Its combination with hesperidin has been proven to be extremely helpful in the treatment of CVI and hemorrhoids, making it a good example of drug synergism. Its interaction with other flavonoids or phytochemicals could be investigated in the future using various methodologies, including system-level knowledge [77,89,90,91,92]. Furthermore, it could also be explored by generating, analyzing, exploring, and integrating large-scale datasets to understand the potentials of Diosmin in case of human diseases [72,73,74,76,77,78,79,90,93,94,95,96,97,98,99,100].

## 7. Conclusions

We have presented a review work that summarizes Diosmin’s pharmacology, significant biological features, such as anti-oxidant, anti-inflammatory, anti-cancer, anti-diabetic, antibacterial, anti-cardiovascular, liver protection, and neuroprotection, therapeutic strategy, possible Diosmin targets, and pathways connected with Diosmin (Figure 1) due to the diverse and potential biological and pharmacological properties and influences of Diosmin (Figure 1). Finally, we have summarized our review work where Diosmin actively plays critical roles in controlling well-known signaling components and the pathways. In this table, we clearly see that there are a number of proteins and these proteins are dominantly associated with inflammatory processes, cancer-associated pathways, antidiabetic, antioxidant, and antibacterial properties.

## Figures and Tables

**Table 1:** Diosmin and its potential interactors followed by their pathways.

Diosmin	NOS2A	Arginine_and_proline_metabolism
Diosmin	NOS2A	Calcium pathway
Diosmin	NOS2A	Long-term_depression
Diosmin	NOS2A	Small_cell_lung_cancer
Diosmin	PTGS2	Arachidonic_acid_metabolism
Diosmin	PTGS2	VEGF pathway
Diosmin	PTGS2	Leishmaniasis
Diosmin	PTGS2	Pathways_in_cancer
Diosmin	PTGS2	Small_cell_lung_cancer
Diosmin	MMP2	Leukocyte_transendothelial_migration
Diosmin	MMP2	GnRH pathway
Diosmin	MMP2	Pathways_in_cancer
Diosmin	MMP2	Bladder_cancer
Diosmin	ICAM1	Cell_adhesion_molecules_(CAMs)
Diosmin	ICAM1	Natural_killer_cell_mediated_cytotoxicity
Diosmin	ICAM1	Leukocyte_transendothelial_migration
Diosmin	ICAM1	Viral_myocarditis
Diosmin	COMT	Steroid_hormone_biosynthesis
Diosmin	COMT	Tyrosine_metabolism
Diosmin	JAK2	Chemokine pathway
Diosmin	JAK2	Jak-STAT pathway
Diosmin	JAK2	Adipocytokine pathway
Diosmin	JAK2	Leishmaniasis
Diosmin	MMP9	Leukocyte_transendothelial_migration
Diosmin	MMP9	Pathways_in_cancer
Diosmin	MMP9	Bladder_cancer
Diosmin	NFKB1	MAPK pathway
Diosmin	NFKB1	Chemokine pathway
Diosmin	NFKB1	Apoptosis
Diosmin	NFKB1	Toll-like_receptor pathway
Diosmin	NFKB1	NOD-like_receptor pathway
Diosmin	NFKB1	RIG-I-like_receptor pathway
Diosmin	NFKB1	Cytosolic_DNA-sensing_pathway
Diosmin	NFKB1	T_cell_receptorpathway
Diosmin	NFKB1	B_cell_receptorpathway
Diosmin	NFKB1	Neurotrophin pathway
Diosmin	NFKB1	Adipocytokine pathway
Diosmin	NFKB1	Epithelial_cell_signaling_in_Helicobacter_pylori_infection
Diosmin	NFKB1	Leishmaniasis
Diosmin	NFKB1	Chagas_disease
Diosmin	NFKB1	Pathways_in_cancer
Diosmin	NFKB1	Pancreatic_cancer
Diosmin	NFKB1	Prostate_cancer
Diosmin	NFKB1	Chronic_myeloid_leukemia
Diosmin	NFKB1	Acute_myeloid_leukemia
Diosmin	NFKB1	Small_cell_lung_cancer
Diosmin	IL2	Cytokine-cytokine_receptor_interaction
Diosmin	IL2	T_cell_receptor pathway
Diosmin	IL2	Type_I_diabetes_mellitus
Diosmin	CDKN1B	ErbB pathway
Diosmin	CDKN1B	Cell_cycle
Diosmin	CDKN1B	Pathways_in_cancer
Diosmin	CDKN1B	Prostate_cancer
Diosmin	CDKN1B	Chronic_myeloid_leukemia
Diosmin	CDKN1B	Small_cell_lung_cancer
Diosmin	PIK3CA	Inositol_phosphate_metabolism
Diosmin	PIK3CA	ErbB pathway
Diosmin	PIK3CA	Chemokine pathway
Diosmin	PIK3CA	Phosphatidylinositol_signaling_system
Diosmin	PIK3CA	mTOR pathway
Diosmin	PIK3CA	Apoptosis
Diosmin	PIK3CA	Focal_adhesion
Diosmin	PIK3CA	Toll-like_receptor pathway
Diosmin	PIK3CA	Jak-STAT pathway
Diosmin	PIK3CA	Natural_killer_cell_mediated_cytotoxicity
Diosmin	PIK3CA	T_cell_receptor pathway
Diosmin	PIK3CA	B_cell_receptor pathway
Diosmin	PIK3CA	Fc_epsilon_RI pathway
Diosmin	PIK3CA	Fc_gamma_R-mediated_phagocytosis
Diosmin	PIK3CA	Leukocyte_transendothelial_migration
Diosmin	PIK3CA	Neurotrophin pathway
Diosmin	PIK3CA	Regulation_of_actin_cytoskeleton
Diosmin	PIK3CA	Insulinpathway
Diosmin	PIK3CA	Progesterone-mediated_oocyte_maturation
Diosmin	PIK3CA	Type_II_diabetes_mellitus
Diosmin	PIK3CA	Aldosterone-regulated_sodium_reabsorption
Diosmin	PIK3CA	Bacterial_invasion_of_epithelial_cells
Diosmin	PIK3CA	Chagas_disease
Diosmin	PIK3CA	Pathways_in_cancer
Diosmin	PIK3CA	Colorectal_cancer
Diosmin	PIK3CA	Renal_cell_carcinoma
Diosmin	PIK3CA	Pancreatic_cancer
Diosmin	PIK3CA	Endometrial_cancer
Diosmin	PIK3CA	Glioma
Diosmin	PIK3CA	Prostate_cancer
Diosmin	PIK3CA	Melanoma
Diosmin	PIK3CA	Chronic_myeloid_leukemia
Diosmin	PIK3CA	Acute_myeloid_leukemia
Diosmin	PIK3CA	Small_cell_lung_cancer
Diosmin	PIK3CA	Non-small_cell_lung_cancer
Diosmin	CDKN1A	ErbB pathway
Diosmin	CDKN1A	Cell_cycle
Diosmin	CDKN1A	Pathways_in_cancer
Diosmin	CDKN1A	Glioma
Diosmin	CDKN1A	Prostate_cancer
Diosmin	CDKN1A	Melanoma
Diosmin	CDKN1A	Bladder_cancer
Diosmin	CDKN1A	Chronic_myeloid_leukemia
Diosmin	IL1B	MAPK pathway
Diosmin	IL1B	Cytokine-cytokine_receptor_interaction
Diosmin	IL1B	Apoptosis
Diosmin	IL1B	Toll-like_receptor pathway
Diosmin	IL1B	Hematopoietic_cell_lineage
Diosmin	IL1B	Type_I_diabetes_mellitus
Diosmin	IL1B	Alzheimer’s_disease
Diosmin	BECN1	Regulation_of_autophagy
Diosmin	CASP9	Apoptosis
Diosmin	CASP9	VEGF pathway
Diosmin	CASP9	Alzheimer’s_disease
Diosmin	CASP9	Parkinson’s_disease
Diosmin	CASP9	Amyotrophic_lateral_sclerosis_(ALS)
Diosmin	CASP9	Huntington’s_disease
Diosmin	CASP9	Pathways_in_cancer
Diosmin	CASP9	Colorectal_cancer
Diosmin	CASP9	Pancreatic_cancer
Diosmin	CASP9	Endometrial_cancer
Diosmin	CASP9	Prostate_cancer
Diosmin	CASP9	Small_cell_lung_cancer
Diosmin	CASP9	Non-small_cell_lung_cancer
Diosmin	CASP9	Viral_myocarditis
Diosmin	IL6	Cytokine-cytokine_receptor_interaction
Diosmin	IL6	Toll-like_receptor pathway
Diosmin	IL6	Hematopoietic_cell_lineage
Diosmin	IL6	Pathways_in_cancer
Diosmin	TP53	MAPK pathway
Diosmin	TP53	Cell_cycle
Diosmin	TP53	Apoptosis
Diosmin	TP53	Wnt pathway
Diosmin	TP53	Neurotrophin pathway
Diosmin	TP53	Pathways_in_cancer
Diosmin	TP53	Colorectal_cancer
Diosmin	TP53	Pancreatic_cancer
Diosmin	TP53	Endometrial_cancer
Diosmin	TP53	Glioma
Diosmin	TP53	Prostate_cancer
Diosmin	TP53	05216_Thyroid_cancer
Diosmin	TP53	05217_Basal_cell_carcinoma
Diosmin	TP53	Melanoma
Diosmin	TP53	Bladder_cancer
Diosmin	TP53	Chronic_myeloid_leukemia
Diosmin	TP53	Small_cell_lung_cancer
Diosmin	TP53	Non-small_cell_lung_cancer
Diosmin	PIK3R1	ErbB pathway
Diosmin	PIK3R1	Chemokine pathway
Diosmin	PIK3R1	Phosphatidylinositol_signaling_system
Diosmin	PIK3R1	mTOR pathway
Diosmin	PIK3R1	Apoptosis
Diosmin	PIK3R1	Focal_adhesion
Diosmin	PIK3R1	Toll-like_receptor pathway
Diosmin	PIK3R1	Jak-STATpathway
Diosmin	PIK3R1	Natural_killer_cell_mediated_cytotoxicity
Diosmin	PIK3R1	T_cell_receptor pathway
Diosmin	PIK3R1	B_cell_receptor pathway
Diosmin	PIK3R1	Fc_epsilon_RI pathway
Diosmin	PIK3R1	Fc_gamma_R-mediated_phagocytosis
Diosmin	PIK3R1	Leukocyte_transendothelial_migration
Diosmin	PIK3R1	Neurotrophinpathway
Diosmin	PIK3R1	Regulation_of_actin_cytoskeleton
Diosmin	PIK3R1	Insulinpathway
Diosmin	PIK3R1	Progesterone-mediated_oocyte_maturation
Diosmin	PIK3R1	Type_II_diabetes_mellitus
Diosmin	PIK3R1	Aldosterone-regulated_sodium_reabsorption
Diosmin	PIK3R1	Bacterial_invasion_of_epithelial_cells
Diosmin	PIK3R1	Chagas_disease
Diosmin	PIK3R1	Pathways_in_cancer
Diosmin	PIK3R1	Colorectal_cancer
Diosmin	PIK3R1	Renal_cell_carcinoma
Diosmin	PIK3R1	Pancreatic_cancer
Diosmin	PIK3R1	Endometrial_cancer
Diosmin	PIK3R1	Glioma
Diosmin	PIK3R1	Prostate_cancer
Diosmin	PIK3R1	Melanoma
Diosmin	PIK3R1	Chronic_myeloid_leukemia
Diosmin	PIK3R1	Acute_myeloid_leukemia
Diosmin	PIK3R1	Small_cell_lung_cancer
Diosmin	PIK3R1	Non-small_cell_lung_cancer
Diosmin	CDKN2A	Cell_cycle
Diosmin	CDKN2A	Pathways_in_cancer
Diosmin	CDKN2A	Pancreatic_cancer
Diosmin	CDKN2A	Glioma
Diosmin	CDKN2A	Melanoma
Diosmin	CDKN2A	Bladder_cancer
Diosmin	CDKN2A	Chronic_myeloid_leukemia
Diosmin	CDKN2A	Non-small_cell_lung_cancer
Diosmin	VCAM1	Cell_adhesion_molecules_(CAMs)
Diosmin	VCAM1	Leukocyte_transendothelial_migration
Diosmin	CASP3	MAPK pathway
Diosmin	CASP3	Apoptosis
Diosmin	CASP3	Natural_killer_cell_mediated_cytotoxicity
Diosmin	CASP3	Alzheimer’s_disease
Diosmin	CASP3	Parkinson’s_disease
Diosmin	CASP3	Amyotrophic_lateral_sclerosis_(ALS)
Diosmin	CASP3	Huntington’s_disease
Diosmin	CASP3	Epithelial_cell_signaling_in_Helicobacter_pylori_infection
Diosmin	CASP3	Pathways_in_cancer
Diosmin	CASP3	Colorectal_cancer
Diosmin	CASP3	Viral_myocarditis
Diosmin	STAT3	Chemokine pathway
Diosmin	STAT3	Jak-STAT pathway
Diosmin	STAT3	Adipocytokine pathway
Diosmin	STAT3	Pathways_in_cancer
Diosmin	STAT3	Pancreatic_cancer
Diosmin	STAT3	Acute_myeloid_leukemia
Diosmin	BCL2	Apoptosis
Diosmin	BCL2	Focal_adhesion
Diosmin	BCL2	Amyotrophic_lateral_sclerosis_(ALS)
Diosmin	BCL2	Pathways_in_cancer
Diosmin	BCL2	Colorectal_cancer
Diosmin	BCL2	Prostate_cancer
Diosmin	BCL2	Small_cell_lung_cancer
Diosmin	BCL2	Protein_processing_in_endoplasmic_reticulum
Diosmin	NFKB1	Ras_signaling
Diosmin	PIK3CA	Ras_signaling
Diosmin	PIK3R1	Ras_signaling
Diosmin	PIK3CA	Rap1_signaling
Diosmin	PIK3R1	Rap1_signaling
Diosmin	BECN1	Apelin_signaling
Diosmin	ICAM1	NF-kappa_B_signaling
Diosmin	IL1B	NF-kappa_B_signaling
Diosmin	NFKB1	NF-kappa_B_signaling
Diosmin	PTGS2	NF-kappa_B_signaling
Diosmin	BCL2	NF-kappa_B_signaling
Diosmin	VCAM1	NF-kappa_B_signaling
Diosmin	ICAM1	TNF_signaling
Diosmin	IL1B	TNF_signaling
Diosmin	IL6	TNF_signaling
Diosmin	MMP9	TNF_signaling
Diosmin	NFKB1	TNF_signaling
Diosmin	PIK3CA	TNF_signaling
Diosmin	PIK3R1	TNF_signaling
Diosmin	PTGS2	TNF_signaling
Diosmin	VCAM1	TNF_signaling
Diosmin	CASP3	TNF_signaling
Diosmin	CDKN1A	HIF-1_signaling
Diosmin	CDKN1B	HIF-1_signaling
Diosmin	IL6	HIF-1_signaling
Diosmin	NFKB1	HIF-1_signaling
Diosmin	PIK3CA	HIF-1_signaling
Diosmin	PIK3R1	HIF-1_signaling
Diosmin	BCL2	HIF-1_signaling
Diosmin	STAT3	HIF-1_signaling
Diosmin	CDKN1A	FoxO_signaling
Diosmin	CDKN1B	FoxO_signaling
Diosmin	IL6	FoxO_signaling
Diosmin	PIK3CA	FoxO_signaling
Diosmin	PIK3R1	FoxO_signaling
Diosmin	STAT3	FoxO_signaling
Diosmin	PIK3CA	Phospholipase_D_signaling
Diosmin	PIK3R1	Phospholipase_D_signaling
Diosmin	NFKB1	Sphingolipid_signaling
Diosmin	PIK3CA	Sphingolipid_signaling
Diosmin	PIK3R1	Sphingolipid_signaling
Diosmin	BCL2	Sphingolipid_signaling
Diosmin	TP53	Sphingolipid_signaling
Diosmin	NFKB1	cAMP_signaling
Diosmin	PIK3CA	cAMP_signaling
Diosmin	PIK3R1	cAMP_signaling
Diosmin	CDKN1A	PI3K-Akt_signaling
Diosmin	CDKN1B	PI3K-Akt_signaling
Diosmin	IL2	PI3K-Akt_signaling
Diosmin	IL6	PI3K-Akt_signaling
Diosmin	JAK2	PI3K-Akt_signaling
Diosmin	NFKB1	PI3K-Akt_signaling
Diosmin	BCL2	PI3K-Akt_signaling
Diosmin	TP53	PI3K-Akt_signaling
Diosmin	CASP9	PI3K-Akt_signaling
Diosmin	PIK3CA	AMPK_signaling
Diosmin	PIK3R1	AMPK_signaling
Diosmin	JAK2	pathways_regulating_pluripotency_of_stem_cells
Diosmin	PIK3CA	pathways_regulating_pluripotency_of_stem_cells
Diosmin	PIK3R1	pathways_regulating_pluripotency_of_stem_cells
Diosmin	STAT3	pathways_regulating_pluripotency_of_stem_cells
Diosmin	PTGS2	Retrograde_endocannabinoid_signaling
Diosmin	IL1B	Inflammatory_mediator_regulation_of_TRP_channels
Diosmin	PIK3CA	Inflammatory_mediator_regulation_of_TRP_channels
Diosmin	PIK3R1	Inflammatory_mediator_regulation_of_TRP_channels
Diosmin	IL1B	Osteoclast_differentiation
Diosmin	NFKB1	Osteoclast_differentiation
Diosmin	PIK3CA	Osteoclast_differentiation
Diosmin	PIK3R1	Osteoclast_differentiation
Diosmin	NFKB1	Longevity_regulating_pathway
Diosmin	PIK3CA	Longevity_regulating_pathway
Diosmin	PIK3R1	Longevity_regulating_pathway
Diosmin	TP53	Longevity_regulating_pathway
Diosmin	IL1B	Hematopoietic_cell_lineage
Diosmin	IL6	Hematopoietic_cell_lineage
Diosmin	PIK3CA	04611_Platelet_activation
Diosmin	PIK3R1	04611_Platelet_activation
Diosmin	PTGS2	Ovarian_steroidogenesis
Diosmin	MMP2	Estrogen pathway
Diosmin	MMP9	Estrogen pathway
Diosmin	PIK3CA	Estrogen pathway
Diosmin	PIK3R1	Estrogen pathway
Diosmin	JAK2	Prolactin pathway
Diosmin	NFKB1	Prolactin pathway
Diosmin	PIK3CA	Prolactin pathway
Diosmin	PIK3R1	Prolactin pathway
Diosmin	STAT3	Prolactin pathway
Diosmin	CDKN1A	Oxytocin pathway
Diosmin	PTGS2	Oxytocin pathway
Diosmin	PIK3CA	Thyroid_hormone pathway
Diosmin	PIK3R1	Thyroid_hormone pathway
Diosmin	TP53	Thyroid_hormone pathway
Diosmin	CASP9	Thyroid_hormone pathway
Diosmin	BCL2	Adrenergic_signaling_in_cardiomyocytes

## Data Availability

Not applicable.
